# Traditional use of the Andean flicker (*Colaptes rupicola*) as a galactagogue in the Peruvian Andes

**DOI:** 10.1186/1746-4269-2-23

**Published:** 2006-05-06

**Authors:** Steve Froemming

**Affiliations:** 1 Buztindegi, 20213 Idiazabal, Gipuzkoa, Spain

## Abstract

This paper explores the use of the dried meat and feathers of the Andean Flicker (*Colaptes rupicola*) to increase the milk supply of nursing women and domestic animals in the Andes. The treatment is of preColumbian origin, but continues to be used in some areas, including the village in the southern Peruvian highlands where I do ethnographic research. I explore the factors giving rise to and sustaining the practice, relate it to other galactagogues used in the Andes and to the use of birds in ethnomedical and ethnoveterinary treatments in general, and situate it within the general tendency in the Andes and elsewhere to replicate human relations in the treatment of valuable livestock. The bird's use as a galactagogue appears to be motivated by both metaphorical associations and its perceived efficacy, and conceptually blends human and animal healthcare domains.

## Introduction

*Hak'achu *is the Quechua name for the Andean flicker (*Colaptes rupicola*) in Ccachín, an agropastoral community in the southern Peruvian highlands. The bird is found primarily in high, rocky pastures and is known for the holes it bores in cliffs and abandoned adobe walls [1]. Other Quechua names for the bird in the southern Andes are *lachu*, *akakllu*, *hak'akllu *and *pitu*. In Aymara, it is known as *yarakaka*.

The feathers and flesh of the *hak'achu *are believed to stimulate milk production. The birds are snared and their carcasses are dried and set aside for later use in ethnoveterinary and ethnomedical treatments. When available – as the bird is neither found nor caught in great numbers – the dried meat is toasted, powdered, mixed with toasted cornmeal, mint, and fennel, and served with salt to the herds. The concoction was often used by my host family in the village to treat their cows. *Hak'achu *is likewise toasted, ground, and served in a broth to women who have problems nursing their infants, and it is given to sheep, goats, and dogs for the same purpose. While both feathers and flesh may be used as a galactagogue, the flesh is considered to be more effective.

The villagers of Ccachín gather single feathers as they find them along village trails, and some women trap the birds in the rocky slopes near their herding cabins. The snares I have seen are made from plastic threads removed from old commercial grain sacks, hand spun to make them finer and stronger. The snare is placed at the entry of a bird's den, so that a bird will be trapped as it enters at night. Once the adult is caught, the young may be taken as well. My host family had as many as four dried carcasses stored away at a time. While I'm only aware of women doing the trapping in Ccachín, Venero Gonzales reports that when a woman is at the point of giving birth in some communities, her husband goes out to capture a flicker to prepare the soup [2].

The use of the Andean flicker as a galactagogue is worth investigating for several reasons: a) it is a pre-Columbian practice that has survived to present and been extended to dairying; b) the bird is trapped by women for the exclusive use of women, one of the few animals so taken; c) the flicker's use as a galactagogue raises questions about the factors that gave rise to the practice and continue to sustain it; and d) the practice is found in both ethnomedical and ethnoveterinary contexts and the factors sustaining the practice in the two contexts differ. According to Alves and Rosa, research on the therapeutic use of animals has been neglected compared to plants, but is becoming increasingly relevant to discussions on public health policies and sustainable management of natural resources [3].

My discussion of the use of the Andean flicker as a galactagogue proceeds as follows. I first provide an overview of the substances used as galactagogues in Ccachín and elsewhere in the Andes, point to the Old World and New World origins of these treatments, discuss the roles of metaphorical and correlational thinking in their selection, and emphasize the difficulty of making correlational assessments in certain contexts. Thereafter, I focus first on the ethnomedical uses of the Andean flicker and then on the ethnoveterinary ones. In the section on ethnomedical use, I discuss possible metaphorical associations, practical effects, and psychological and social factors that underlie the practice. In the ethnoveterinary section, I discuss the therapeutic uses of birds in general, the use of salt, protein, and other nutritional supplements for cattle, and Andean peoples' use of the same health care models for their animals as they have for themselves. In the concluding section, I highlight three issues that emerge from the preceding: the origin and cultural transmission of such ethnomedical and ethnoveterinary practices, the underlying practical and symbolic logics, and the conceptual blending of human and domestic animal domains.

I conducted the ethnographic research on which this study is based over a twenty-eight-month period from 1989–1992, with follow-up field work in 1998. The village in which I conducted field work is situated between 2,300 and 4,500 masl in the Department of Cusco, Peru. I gathered much of the information from the family I lived with over this time, the women in particular being knowledgeable on home remedies of all sorts. The family had a large cattle herd, sheep, and alpaca, and was primarily interested in flickers for their ethnoveterinary value. *Compadres *and other key consultants in the village provided further information on the local use of the galactagogues I report on here, and comparative data on the variety of galactagogues used in the region as a whole comes from a review of the Andean literature.

## Background

Health care is moderately pluralistic in Ccachín. Self-care, traditional curers, and Western medicine are all treatment options. The public health worker at the local medical post is increasingly important in family planning and the treatment of wounds and minor illnesses, but the nearest hospital is a half-day's truck trip away, and physicians are an option of last resort. Self-care is the most common treatment choice, with most illnesses treated solely with home remedies or with home remedies in the mix.

### Substances used as galactagogues

Flicker carcasses are one of several treatments used in Ccachín to attempt to increase the milk flow of nursing women and animals. *Apanqora *(crayfish; *Astacus fluvialis*) are sometimes brought back from the lower Lares Valley and mixed with ground corn and salt for the cows in place of *hak'achu*. Commercial fishmeal is a more costly and less used alternative. Women who have just given birth are served a broth of quinoa if they have problems with their milk. Given the choice between *hak'achu *and quinoa, villagers say that *hak'achu *is the best. A broth of *askantuy*, a white larvae found in the central spike of *achupalla *(*Puya herrerae*), is used in many parts of the Andes for the same effect [4,5]. *Askantuy *is used to treat anemia in Ccachín. Nutritional anemia is a problem for both sexes and for all ages, but given that it is especially prevalent among pregnant and nursing women in developing countries [6], its use for anemia and milk production suggests that it is prescribed in overlapping contexts. *Hinojo *(fennel; *Foeniculum vulgare*) is served in a tea to women and added to the salt mixture for cows in Ccachín. Fennel is widely associated with enhanced milk production throughout the Andes.

In other areas of the Andes, *ajonjilí *(sesame seeds; *Sesamum indicum*), *brezo *(heath;* Erica vulgaris*), and a broth of beef lung may be served to help a woman's milk comes in. The eyes of sweet potato (*Ipomoea batatas*) may be ground, soaked in wine, and plastered on a woman's breasts. Just as crayfish are used to augment the milk production of cows in Ccachín, a broth of *yukra *(river shrimp; *Bithynis caementarius*) is used as a galactagogue for women in Arequipa [4,5,7]. North of Ccachín, in the valley of Quebrada, a turkey-like bird, the *manaqaraku *or speckled chachalaca (*Ortalis guttata Spix*), is served in tea or broth to nursing women [8]. Table 1 summarizes the wide variety of substances used as galactagogues in the Andes.

**Table 1:** Galactagogues in the Andes

**Preparation**	**Ingredients**	**Location**
Amulet	Stone or ceramic representation of cheese	Abancay [55]
Beer (Commercial)	Beer (dark or malted)	Callao, Lambayeque, Libertad, Lima [4]
Beer (*Chica*)	Corn	Cusco, Huánuco, Junín, Arequipa [4]
Beer (*Chica*)	Corn with mouse excrement	Huancayo, Junín [4]
Beer (*Chica*)	Barley	South and Central Peru [4]
Beer (*Chica*)	Peanuts	South and Central Peru [4]
Beer (*Chica*)	Quinoa	South and Central Peru [4]
Beverage	Donkey's milk	Huáncayo [4]
Beverage	Honey	Cusco [4]
Beverage	Water used to boil corn	Arequipa, Lima [4]
Cooked	*Arrakacha *greens (*Arracacia esculenta BC*.)	Huánuco [4]
Cooked	Fennel	Huánuco [4, 5]
Cooked	*Manka p'aki *(*Eupatorium peregrinum *or *Eupatorium sternberginianum*, a plant)	Arequipa [4]
Cooked	Quinoa with milk	Yanatile (Cusco) [8]
Cooked	Quinoa with mouse excrement	Junín [4]
Cooked	Rice, chocolate and *chufla*	Northern Peru [4]
Cooked	*Zarandaja *(Val bean; *Dolichos lablab*)	Northern Peru [4]
Massage	Quinoa (cooked)	Cusco [4]
Plaster	Sweet potato eyes and wine	
Roasted	Guinea pig excrement and incense	Cusco [4]
Soup	*Askantuy *larvae (toasted)	Cusco [4, 5]
Soup	Earthworms (toasted)	Southern Peru [4]
Soup	Beef lung	Cusco, Huanuco [4, 7, 8]
Soup	Cow or pig entrails	Callao, Lima, Ica, Libertad [4]
Soup	guinea pig	Southern Ecuador [38]
Soup	*Ch'uñu *(freeze-dried potatoes)	Puno [51]
Soup	*khaya *(freeze-dried oca)	Cuyo Cuyo [56]
Soup	*Hak'achu *(Andean Flicker)	Cusco, Puno [4, 7, 8]
Soup	*oca *(*Oxalis tuberosa*)	Cuyo Cuyo [56]
Soup	*Pijuayo *(a palm)	Loreto [4]
Soup	Quinoa	Cusco, Huánuco, Puno [4, 8, 51]
Soup	Quinoa with beef lung	Cusco [8]
Soup	Quinoa with fennel	Arequipa [4]
Soup	Rice	Puno [51]
Soup	Shrimp	Arequipa [4]
Soup	Soybean	Quebrada (Cusco) [8]
Soup	Vegetables	Cusco [8]
Tea	Anise	Cusco, Cajamarca [4, 5, 8]
Tea	Cotton seed (toasted, ground)	Quebrada (Cusco) [8]
Tea	Fennel seed and roots with honey, celery, and valerian	Cajamarca [8]
Tea	*Manaqaraku *(*Ortalis guttata Spix*., a jungle bird)	Quebrada (Cusco) [8]
Tea	Milk and toasted sesame	Lima [4]
Tea	Sesame seed	Jauja, Junín [4]
Tea	Sweet potato leaf or tuber	Lima, Quebrada (Cusco) [4, 8]
Tea	Wheat (toasted)	Lima [4]
Toasted	Peanuts	Ica [4]

### Old and new world origins of the treatments

Some of the remedies used in the Andes appear to be native to the region, others introduced. In the 16th century, Phayer, writing in Europe, recommended in his "Remedie appropriate to ye encreasing of Mylke the Brestes"

"Parsneppe roots, fenelle roots sodden in broth of chicken and afterwards eaten with a little freshe butter rice soaked in cows milk, powder of earthworms dry and dronken in the broth of a neates tonge, the broth of an old cocke, with myntes, cynanon, and maces." [[9], cited in [10]]

Fennel, powdered earthworms, beef organs, and the broth of certain birds were all used as galactagogues in Europe at the time of Conquest, and it seems that many of these beliefs first took hold in the Andes through a process of diffusion. On the other hand, Cobo, one of the early Spanish chroniclers of the Andes, notes that the flesh of the *hak'achu *was held in esteem by the Inca for stimulating milk production [11]. The use of *hak'achu *appears to be a native practice that has continued unbroken in the Andes for well over five hundred years.

### Metaphorical vs. causal associations

Both metaphorical and causal associations underlie the galactagogues used in the Andes, and sometimes the two coincide.

Jelliffe and Jelliffe have noted that many of the preparations, techniques, and rituals used for enhancing milk production throughout the world seem to be based on the imitative principle of sympathetic magic that "like affects like" [12]. The use of milk-like substances, the administration of liquids to produce a fluid secretion, and the use of charms depicting large breasts are commonplace. The use of amulets representing cheese as galactagogues in the Andes falls into this category, as does apparently the consumption of mouse excrement, though more indirectly, though a connection with cheese (Table 1). The application of sweet potato eyes with wine to the breasts likewise appears to be based on relations of similarity. Many of the treatments used with opposite effect – to suppress milk production at weaning – in the Andes are even more suggestive in this regard, perhaps because they are not so based on diet (see Table 2). Such practices as throwing breast milk into the hearth or out in the sun to dry and using a plaster made from the stem end of a squash (playing off of the fact that the same word – *pezón *– is used for both this part of the squash and nipple in Spanish), are evidently grounded in relations of likeness, the latter suggesting that linguistic relationships sometimes substitute for causal ones as well.

**Table 2:** Techniques used to suppress lactation in the Andes. These remedies are drawn from *La Medicina Popular Peruana: Contribución al Folklore Médico del Perú *by Valdizán and Maldonado, 1922. [4]

**Preparation**	**Location**
Extract the milk and throw it out in the sun	Junín, Huánuco
Throw milk extracted from breasts on the cooking fire	Lambayeque, Piura, Tumbes, Lima, Callao, Ica, Ayacucho
Rub the breasts with ground garlic	Puno
Tie the nipples with a dirty table cloth	Cajamarca
Place cloth soaked in cold water over the breasts	Arequipa
Use preparation of cooked *pezón *(stem end of *zapallo *squash) and *wiñapu *(germinated corn for making beer)	Arequipa
Use water from cleaning the shirt of the father of the child	Arequipa
Use ashes from hair taken from the nape of the neck of the father of the child suspended in wine	Arequipa
Use the powder from ivory dissolved in wine	Arequipa
Use the powder from sealing wax dissolved in wine	Arequipa
Use the blood of a rooster's crest	Lambayeque, Piura, Libertad
Use white clay suspended en boiling water in which a heated steel nail has been added	Apurímac
Use excrement of dove with *wakatay *(*Tagetes minuta*)	Apurímac
Use a watery maceration of the infant's umbilical cord	Huancayo
Eat a large quantity of watercress	Junín

The Andean flicker is commonly referred to as *llulla hak'achu*, the "lying flicker," because of local lore surrounding it's haughty call and red nape. Tradition has it that the *hak'achu *has a red patch on the nape of the neck because it lied and had its tongue ripped out in punishment [13]. Sent by the Creator to announce that fruit could be found in both valley and puna, the *hak'achu *told people that fruit would grow only in the hot valley regions. Sent to announce that the Creator would make twelve colors of sheep so that the poor would not have to buy dye, the *hak'achu *reported that there would be four colors of sheep. Despite the Creator's intentions, whatever the *hak'achu *announced came to pass [13].

Given such associations, feathers of the *hak'achu *are believed to protect people who bear them from envy, and they are used as talismans to protect cattle from theft. While these uses are rather transparently grounded in sympathetic magic, the cultural logic underlying the bird's use in enhancing milk production is not as readily apparent. Does metaphor underlie the use of *hak'achu *broth and other treatments for nursing females listed in Table 1 as well, or are some of these practices functionally explainable in terms of their beneficial consequences? Potentially, both. We cannot discount the possibility that many traditional galactagogues in the Andes have beneficial pharmacological effects that are culturally identified and explained in terms of observed likenesses rather than their biochemical causes. The likenesses are readily accessible to users, the actual causative agents not.

Some young women in Ccachín express doubts about *hak'achu *feathers' effectiveness in protecting their herds from envy. "My mother believes it" one told me, "but I do not." The younger generation doesn't seem to question the value of *hak'achu *in enhancing milk production, however. As villagers are schooled and come to doubt traditional supernatural beliefs in this and other realms, belief in sociomagical and supernatural cause-effects is eroded. Metaphorical cause-effect relations associated with natural processes, however, appear to be much more resilient.

### The difficulty of making correlational assessments

The assumption that people in all societies seek to acquire empirical knowledge about what causes what, what influences what, and what goes with what in their environment is empirically justified and theoretically unproblematic. Shweder [14], however, has persuasively argued that correlation and contingency are relatively difficult and nonintuitive concepts, and that there is a universal inclination to substitute propositions about language for propositions about the world. According to Shweder, people everywhere have difficulty drawing correlational lessons from their experience in some contexts, and substitute meaningful, symbolic connections among objects and events – similarity, synonymy, part-whole relations, function, and origin, collectively referred to as "likeness" or "resemblance" – in their place.

What are these contexts? When is it difficult to make correlation assessments? The information-processing system that has evolved for humans to cope with the complex and probabilistic causal relations of the environments in which we find ourselves is characterized by sequential processing, limited memory, selective perception, and simplifying heuristics. Feedback is central. Action induces feedback, and the monitoring of incremental judgement-action-feedback loops is relied on more than commitment to a priori judgements of the best course of action [15]. The nature of cue-criterion relations is crucial. Women in Ccachín use onion heels and lemon peels to cut grease on pots and pans, for example. Appreciation of the cause-effect relationship is inductively simple and straightforward. This contrasts sharply with the difficulties of controlling the many factors affecting milk production. In complex environments, humans do best when feedback is immediate and frequent, when there is a lot of redundancy between cues, and when observations don't increase or decrease linearly over time, exhibit random fluctuations, or change their characteristics.

Table 3 lists some of the beliefs in Ccachín where metaphoric relations appear to substitute for correlational ones. The substitution of likeness for co-occurrence likelihood is commonplace in traditional indigenous Andean knowledge systems. This is not to say that it is more common than accurate assessment of causality, nor that it is more common and ubiquitous than in everyday thinking in our own society. Rather, it is simply salient from the standpoint of an observer who comes from a society with elaborate and expensive institutional and technical structures for investigating the correlational likelihood of many of the same phenomena, and a formal educational system to disseminate the assessments of "experts" among the general public.

**Table 3:** Some examples of sympathetic magic in Ccachín

**Item**	**Association and Practice**
Miniature Representations	A variety of small stone carvings and naturally-formed stones are used in rituals to represent animals and crops, to communication with the spirits, and to promote fertility and abundance. In addition, dancers from the village construct miniature representations of desired futures during the pilgrimage of Qoyllur Rit'i, along with people from throughout the region.
Left-Spun Yarn	*Lloq'e *(S-twist, left-spun yarn) is believed to protect one from witchcraft and evil winds. Left-spun yarn is used as an antidote to sorcery (*lloq'e *in Quechua means "left"; the verb *lloq'ey *means "to hex"). Most women incorporate a small bit of *lloq'e *into their weavings. It's considered essential in the clothing woven for the inauguration of new community officials.
Black- Chested Buzzard Eagle	The talons of the Black-Chested Buzzard Eagle (*Geranoaetus melanoleucus*) are sometimes braided into the ropes that men make to lasso cattle to improve their aim. The ropes are braided on the "good days" of Monday, Wednesday, Thursday, or Saturday for success.
Coca Reading	The shape, condition, and position of coca leaves relative to each other are used in divination to represent age, sex, relationship, emotional states, illnesses and health, and activities according to metaphoric likenesses. Fright, for example, is represented by serrated edges, and death is represented by the tip broken away from the rest of the leaf as the soul separates from the body.
Twelve Yarns with the Colors of the Rainbow	The rainbows that form around waterfalls, cascades, and springs are believed to cause *k'uychi onqoy *(rainbow sickness) in Ccachín. The rainbow enters a woman's body while she is urinating and causes her abdomen to swell as if she were pregnant. A diagnostic feature is that the urine takes on the colors of the rainbow. To treat it, twelve colors of wool yarn are toasted and ground together (according to my informants, synthetic yarn doesn't work). A tea or broth is made of the powder, and this is given to the patient.
Extraordinary Products from the Earth	*Wanlla*, specimens of agricultural products, minerals, and precious metals with outstanding quality or unusual characteristics, are wrapped with coca leaves and set aside for veneration. They are associated with the *pachamama *(earth mother) and referred to by their specific forms: *saramama *(corn mother) and *papamama *(potato mother), for example. They have the power to engender themselves in abundance.
Coca Wads	*Hach'u*, the wad of chewed coca leaves, is associated with animal manure, and is buried during fertility rituals and first-haircutting ceremonies to enhance the fertility of the herds.

The substitution of conceptual relations for correlational ones may be universal in certain contexts, but selection of particular class inclusion and metaphoric relations as a point of focus often seems arbitrary, and reconstruction of the historical origin and evolution of the beliefs can be subject to the telling of just-so stories. To some extent, however, there appear to be cross-cultural and culturally-specific patterns, and these are worth exploring.

## Ethnomedical use of the Andean flicker

### Metaphorical associations

Is metaphor at play in the use of such things as *hak'achu*, quinoa, and *askantuy *larvae for increasing milk production in Ccachín? If so, what associations are made? Metaphoric connections are specific to the salient features of the items being compared, but taken together, there may be patterns.

One possibility is suggested by the milk-white brew made from *askantuy *larvae. Quinoa broth likewise has a white, creamy appearance. Color enters into humoral classification, and it may be metaphorically important in this context as well. Victor Turner suggests that the colors of red, white, and black universally signify blood, milk or semen, and feces respectively, with perception of the world referenced to what we subjectively know best – the human body [16]. The contrast between white and red in several of the techniques used to suppress lactation in the Andes is intriguing in this regard (Table 2). Color alone, however, would not seem to sufficiently narrow the range of available alternatives. The milky-white secretions of *pampa llank'u *(milkweed), *kiska qhana *(sow thistle), and *wach'anka *(*Euphorbia huachangana*) are also put to medical use in Ccachín, but none of them are believed to enhance a woman's milk production. *Pampa llank'u *is rubbed on one's legs and other extremities when they hurt from the cold, *kiska qhana *is used as a tea for heart pain, and the milky liquid obtained from *wach'anka *makes a purgative used to treat drunkenness and a pesticide for rats and mice. Notably, however, thistle tea is used as a folk remedy for stimulating milk production in the U.S. [10].

A second possibility is that the qualities of wet and dry are salient. The dried *hak'achu *remains are reconstituted and served as broth. Drying and rehydrating are commonplace operations in the Andes. The Inca mummified their deceased rulers, and pre-Incan peoples are still believed to exist through their bones and mummified remains. Dried llama fetuses are "purified by the sun" and served to the mountain spirits [17,18]. Potatoes are frozen and sun-dried as *ch'uñu*, where they are conceived as having an ambiguous "life-in-death" state of being [19]. Meat is sun-dried as *charki *(a Quechua term that has made its way to the English language as "jerky"), and both *ch'uñu *and *charki *are reconstituted in water before serving. Villagers in Ccachín say that one should not throw corncobs out the door because the person who throws them out may swell up in the rain and dry up in the sun as the cobs do. The sympathetic associations have the effect of reinforcing a practical injunction in this case, as corncobs are used as scrub brushes, knife handles, and fuel for fires.

Both *ch'uñu *(freeze-dried potatoes) and *khaya *(freeze-dried oca) served in soups are used in one area or another of the Andes as galactagogues. The act of transforming *ch'uñu*, *hak'achu*, quinoa, and other galactagogues from a dry liminal state to a hydrated, life-giving one may be sympathetically linked to the hoped-for pattern in the breast. In reverse, when a woman needs to suspend lactation, a bit of milk from her breasts is swabbed in a wad of cotton and set out in the sun to dry (Table 2) [4,7]. The logic of dehydration and reconstitution doesn't explain why *hak'achu *carcasses are used as galactagogues rather than those of the *chiwanku *thrush (*Turdus chiguanco*), however, or why quinoa is used instead of wheat or corn, so it cannot provide a full explanation of the practice.

The *hak'achu *is not the only bird dried and used for medicinal purposes in the Ccachín. The Andean condor scavenges carrion and soars on the winds sweeping up the east flank of the Andes. Correspondingly, it is associated with illnesses and death caused by the malevolent *machu wayra *winds (see Table 3). Condor feathers are toasted, ground, and brewed in the same manner as *hak'achu*. The broth or tea is used to treat respiratory diseases. One of my *comadres *made just such a tea from a single condor feather for her aging mother when the latter was gravely ill. Hummingbird carcasses are also dried and used in the Cusco area. With its rapid, blurry movement, the hummingbird is considered to be a messenger bird, usually an omen of death. Allen, for example, recounts an encounter with a hummingbird that was interpreted by her informants as portending the death of her grandmother [19]. A soul may leave the body to visit faraway relatives through the medium of a hummingbird, especially during the eight days that the spirit stays near the family after a person's death. The roasted carcass of the hummingbird is used in treating fright in children and soul loss, and it may be substituted for condor feathers in some treatments when the latter are unavailable [7]. The flesh of the *chiwanku *thrush is used in the Department of Huánuco for jaundice and other liver ailments. A metaphorical association between illness and treatment is suggested in this instance by the frequency with which this bird discharges its excrement [4].

Given these and other associations based on the perceived traits of birds (the use of the talons of black-chested buzzard eagles, for example, is described in Table 3), it seems equally plausible that there is some trait of the Andean flicker that establishes a metaphorical association with milk or lactation. *Hak'achu *flesh is used as a meat in soups and stews in the Cusco area [7]. *Hak'achu *blood is mixed with wine to treat heart problems, dropped in the ear to counter hearing loss caused by the wind, and taken as a drink to combat tuberculosis [4,7,13]. The feathers are used for cleaning teeth [7]. In some areas of Cusco, a *hak'achu*'s tongue is carried as a charm to help one find love [13]. In Andean lore, the *hak'achu *carries the leaf of a secret plant in its beak to soften rock while boring its nest [7,13,20]. Perhaps because of this tenderizing effect, it's said that a woman pecked by the beak of a flicker will thereby become an adept and appreciated cook.

Tradition has it that the Inca learned the secret of this herb from watching the *hak'achu*, and applied it in their monumental architecture [13]. In the 17th century, Padre Antonio de la Calancha wrote that thieves and delinquents at Potosí (Bolivia) used the herb to dissolve stone and metal bars to escape from the jails [13]. Venero Gonzales [2] reports that the plant is often said to be *manka p'aki *(*Eupatorium peregrinum, Eupatorium sternberginianum*), which translates as "broken pot," further extending the metaphorical connections (*manka p'aki *also is used as a galactagogue in its own right in the Arequipa area). I suspect that this association of the bird with a magic herb traveled with the Spanish from Europe, where certain woodpeckers were believed to use moonwort (a fern) or "springwort" (an herb unknown to humans) to dissolve the materials people used to plug their nests with. The springwort legend appears to originate even further east, where it appears in the Arabian Nights, the Koran, and the Talmud [21]. The association could have been reinvented in various cultures many times, however, from watching the behavior of the birds.

While there are several kinds of woodpeckers in the Andes, they have enough in common that beliefs seem to pass readily across species boundaries. Elsewhere in Peru, the heart of a woodpecker is added to wine to treat epilepsy, and the feathers are used in treatments to prevent spontaneous abortion and facilitate delivery [4]. The association between woodpeckers and childbirth is notable, and the metaphoric connection to softening objects and opening/closing passages is clear.

The association between *hak'achu *and milk production has not been explained by my informants, for whom it is common knowledge and taken as a given, and the logic is not obvious to an outside observer from the characteristics of the bird. The bird's tongue, red nape, audible call, association with rocky terrain, and habit of making holes are the features highlighted in stories about the bird. The *hak'achu *favors the high puna grasslands where people pasture their cattle, although it's not the only bird to do so. The association with cattle, however, extends as far south as Argentina, where it is likewise considered to be the enemy of cattle rustlers. There may be a good practical reason: it is said that when thieves move among the herds, it alarms the birds and their calls warn the herders [22].

While humoral medicine is practiced in Ccachín with both humans and animals, and the hot/cold classification system central to it in part is metaphorically derived, I never heard the effectiveness of *hak'achu *explained in terms of its hot/cold properties. Several of the criteria used in hot/cold classification in the Andes would potentially apply to the use of flicker remains as a galactagogue, and it remains to be seen if it is interpreted in such terms in other Andean communities. The hot-cold classification system has its origins in the humoral theory of the Old World, where it was the protoscientific doctrine of the medical establishment at the time of the Spanish conquest, and it continues to be practiced throughout Latin America, especially in the mountain regions [23-25]. Ethnomedical practices in the Andes have various origins, both pre- and post-conquest, and many of these bits and pieces have gradually been integrated into the humoral model, serendipitously in some instances, assimilated by the model in others [24]. But there is no attempt to integrate many practices, and they maintain a logic all their own.

### Practical effects

Metaphorical associations aside, the question remains whether the techniques used in Ccachín to stimulate the flow of breast milk have the consequences ascribed to them, and if so, whether they are more likely to lead to the desired outcome than available alternatives.

Hundreds of plants are used as galactagogues worldwide, but the biochemical actions of only a few of them have yet been investigated. Some of those investigated have been found to have estrogenic, oxytocic, or other hormonal effects in lab conditions [26]. Notably, the pharmacologically-active constituents of fennel – sometimes added to the corn-salt mix given to cows in Ccachín to promote milk production and used for the same purpose by women in some areas of Peru – are dianethole and photoanethole, polymers of anethole. Anethole is structurally similar to the catecholamines, which are believed to influence secretion of prolactin [27]. In one study, fennel oil increased milk production and the milk's fat content for goats [28]. On the other hand, mint (*Mentha spp*), also sometimes added to the corn-salt mix given to cattle in Ccachín, may reduce or dry up the milk supply in both humans and animals when taken in sufficient quantities [29,30].

While there are not many data on the incidence of lactation failure in humans, it is considered to be rare, especially relative to the amount of cultural concern given to it in many societies [12,31]. The critical importance of successful breast-feeding through human history has apparently selected against poor milk producers [12]. The lactation process involves mother-infant interaction, and failure may be caused at either end. The infant's sucking affects the quantity of milk produced, and a variety of sucking and swallowing disorders (prematurity, cleft palate, etc.) can affect milk production. Given the importance of sucking, some have suggested that the spread of bottle-feeding and widely-spaced, scheduled breast feedings has led to an increased incidence of "insufficient milk syndrome" – real or perceived milk insufficiency [32]. This pattern is most common in industrialized urban areas, but unlikely in Ccachín, where the single case of bottle-feeding I witnessed was for an infant whose mother had died soon after childbirth. There are anatomical, hormonal, nutritional, and pharmacological causes that can affect both the production and release of milk, but the literature has predominantly emphasized psychosocial interference with the release of milk, the so-called "let-down reflex" [31].

While unknown pharmacological effects of some of the galactagogues used in Ccachín can't be discounted, the effects of *hak'achu *broths, quinoa broths, and others as fluid supplements, as nutrient supplements, and as reassurance on the let-down reflex are the most probable and the best understood.

A positive relationship between milk production and drinking fluids is often hypothesized. It's also backed by anecdotal evidence. A woman I rented a room from in Cusco, for example, reported noticing a difference in her own milk flow depending on her fluid intake. Various investigators, however, have found that variation in water intake within wide limits has no physiological effect on the volume of milk produced, and this is consistent with the anti-diuretic effects of prolactin, one of the principal controlling pituitary hormones [12]. The widespread belief that fluid intake affects milk yields emerged either as a response to extreme conditions (severe enough to affect health in general), or is another instance of likeness substituting for correlational likelihood (fluid in, fluid out). As Orlove notes, the rural Andean practice of serving boiled soups and stews in the early morning and late afternoon has the adaptive benefit of providing sufficient water to avoid the risks of dehydration in the dry season, while reducing the danger of ingesting parasites from contaminated water sources [33]. These preferences seem generally sufficient in themselves to ensure that a nursing mother receives enough fluids. In any case, the belief in the importance of increased fluid intake while lactating does not explain why particular broths are considered more effective in stimulating milk production than others.

A common thread linking many of the galactagogues in Ccachín is their high protein content. Quinoa averages 16% protein by weight, compared to 8–12% for corn, the staple cereal in Ccachín. Its protein quality is close to the FAO standard, comparing well with milk and meat. Quinoa is high in such essential amino acids as lysine and tryptophan [34]. Corn, on the other hand, is notably deficient in these limiting amino acids. *Hak'achu *has been hunted for its meat since Incan times, if not before, though in Ccachín its use is exceptional and it is not thought of as a game dish. *Askantuy *larvae are used medicinally rather than as a food source, but another larvae, *wayt'ampu *(*Metardaris cosinga*) is recognized for its nutritional value and is said to be a brain food [35].

The maternal causes of lactation failure and the effect of dietary supplements are not well understood. Studies of the lactation performance of both well-nourished and malnourished women in recent years have found a surprising consistency in daily milk volumes, and it appears that physiological mechanisms exist to compensate for inadequate maternal nutrient intake, with cumulative long-term nutritional cost born by the mother [36]. The threshold level below which a woman is unable to produce a normal quantity of milk appears to be quite low. One study in Gambia found that there was no significant decrease in milk volume when caloric intake fell to 1200 kcal/day, 70% of the women's average consumption. One of the best studies we have of the seasonal fluctuation in caloric intake in the Andes, conducted in Nuñoa (Puno) in 1985, shows a fluctuation of between 1250 calories pre-harvest and 1900 calories post-harvest for women in their childbearing years [37]. This is above the 1200 kcal/day threshold, though whether this threshold holds in a high mountain environment is an open question. On the other hand, studies in Nigeria and India have shown significant increases in milk output when women are given a protein supplement increasing their protein intake from 50–60 grams/day to 100 grams/day [36]. According to one of the most comprehensive nutritional studies we have for the Southern and Central Andes, the *Encuesta Nacional de Consumo de Alimientos *of 1971–1972, annual per capita calorie and protein consumption for households that consumed primarily traditional foods varied from 2045 calories and 56 grams of protein/person/day for low income households to 2716 calories and 83 grams of protein/person/day for high income households [33]. However, these statistics are for adults of both sexes combined, and valuable as they are, a one-year study does not adequately capture the susceptibility of highland populations to environmental fluctuations.

As with the quantity of protein, the quality of protein and non-protein nitrogen compounds in the milk may be affected by maternal nutrition under threshold conditions, but the effect seems to be minor in magnitude. Most studies show that the protein content of human milk is relatively constant regardless of maternal nutrition [36]. Overall then, existing research on the effect of maternal nutrition on milk volume is inconclusive, with limited evidence that substantial protein supplements can affect milk production and supplements of protein and vitamins can affect milk quality under certain conditions. Given our current knowledge, it seems unlikely that traditional high-protein food supplements for nursing mothers in the Andes can be related to any observed increases in milk production, except perhaps at certain undetermined threshold conditions. Such supplements, of course, promote good nutrition, and in so doing, prevent illness, fatigue, and general states of unwellness that can stress and strain long-term nursing. The cumulative deterioration of nutritional status through maternal depletion can shorten the lactation period [12].

Unlike areas of the world where dietary taboos while nursing severely limit a woman's protein intake, dietary practices in the Andes contribute to a mother's general health. Except for quinoa, the local grain with the highest protein content, however, it's hard to explain the specificity of some of the more exotic local galactagogues used as protein supplements. Any domestic or game animal protein should do, and unlike *hak'achu *and *askantuy*, many of the alternatives are available in significant quantities. Hirschkind reports that chicken is an indispensable ingredient in the soup prepared during the forty-day post-partum diet used by women in Cañar province of the southern Ecuadorian highlands, and guinea pig soup is consumed by the women of that area during lactation to help them produce plenty of milk as well [38]. Chickens and guinea pigs are not only very ready sources of protein in Ccachín and many other rural villages in Peru as well, they are raised primarily by women. On the other hand, neither *hak'achu *nor *askantuy *are consumed as standard fare, and the beliefs about their value as galactagogues may have emerged under conditions of environmental stress.

### Psychological and social factors

Under normal conditions, the probable explanation for the positive effect of most milk-producing treatments in the Andes is psychophysiological. Indeed, among health domains, lactation is a sphere where social and psychological factors play an exceptional role. Emotional responses appear to play a key role in the success and failure of lactation in both nursing mothers and dairy animals; so much so that lactation has sometimes been termed a "confidence trick" [12]. Apprehension and anxiety inhibit milk secretion by interfering with the let-down reflex. Sufficient milk is available, but it doesn't reach the infant. Emotional upset triggers a vicious cycle as lactation failure increases anxiety and doubt during subsequent feedings. At the same time, breast engorgement interferes with an infant's ability to suckle and poor milk drainage increases the probability of bacterial infection, all further contributing to lactation failure. The consensus of contemporary Western medical opinion seems to be that most galactagogues work as confidence inducers, reinforcing the let-down reflex.

High-protein animal products are coveted in Ccachín, and the etiquette of their meal-time distribution can influence one's self-esteem and emotional well-being, beyond their positive nutritional effects. People often talk about who gets the most and the best portions of meat in Ccachín. *Hak'achu *flesh and some other local galactagogues are not served in sufficient quantities to have much impact on general nutritional levels, but have an advantage over more mundane protein sources for emotional impact in their exceptional character. Their specialness and rarity single them out. A parallel can be drawn here to the use of woodpecker-scalp headbands by natives of Northwest California in their Jumping Dance [39]. Such headbands were regarded as treasures and used in shamans' fees, bride prices, injury compensations, and other exchanges. The rarity of the scalps contributed to their value, and the dance gave their owners an opportunity for their public display. The expressive and sociocultural characteristics of the bird's use as a galactagogue could help explain any psychophysiological effectiveness that it has for human populations.

## Ethnoveterinary use of the Andean flicker

Psychophysiological effectiveness, however, cannot explain the use of the flesh of the Andean flicker as a galactagogue for livestock. Not that dairy animals are not subject to some of the same emotional variability that humans are. Much of what we know about the psychophysiology of lactation comes from the folk experience of dairy farmers and from modern dairy science. Around the world, herders have recognized the effect that individual temperaments, responses to other members of the herd, being spooked by predators, being milked by strangers, and changes in daily patterns can have on the milk production of their animals. But there's no reason to suppose that adding a bit of *hak'achu *flesh, crayfish, or fishmeal to the monthly salt ration has any let-down effect. If these ethnoveterinary practices have the effect they are believed to have, it is because of their nutritional or pharmacological value, not because of an effect on the psychological well-being of the animals.

### The use of birds in ethnoveterinary treatments

There are other examples of birds being used in ethnoveterinary treatments worldwide. Adolph, Blakeway, and Linquist report that among the Dinka and Nuer of East Africa, vultures are the main ingredient cooked in a soup given to sick cattle [40]. They speculate that just as chicken soup apparently has a clumping effect on leukocytes which explains its value as a traditional common cold remedy [41], vulture soup may do the same. The aforementioned role of chicken soup in the post-partum diet prescribed for women in the southern Ecuadorian highlands is notable in this regard, as is the use of the turkey-like *manaqaraku *(speckled chachalaca; *Ortalis guttata spix*.) in a tea or broth as a galactagogue for women in the jungle areas of Peru. Beyond the general importance of health for lactation, there is a difference between treating respiratory infections and boosting milk production, however, and the remedies are not necessarily interchangeable. One need be cautious about reference to such purported pharmacological effects, but they cannot be ruled out without testing.

Earlier, I noted that a broth of condor feathers is used in Ccachín to treat respiratory infections, much like vulture soup is used to treat such illnesses in East Africa. The similar treatments may be explained by similar metaphorical associations, with both birds soaring on the winds, or by similar biochemical effects as Adolph, Blakeway, and Linquist suggest, but given the specificity and characteristics of the birds associated, out of a seemingly wide range of functional equivalents, metaphor probably carries the explanatory weight. Chickens, which were introduced to the Andes with the Spanish conquest, are far commoner in Ccachín than condors. The Andean condor is autochthonous, and its use in treating respiratory infections in the Andes thus has greater antiquity. In terms of functional equivalence, such historical contingencies may also help explain the preference for one over another.

### Nutritional supplements for cattle

Because of the distance of the pastures in Ccachín, salt is given to the cows every four to six weeks. As noted earlier, about twenty-five pounds of corn is toasted, ground, and added to this mixture, sometimes with a few herbs. When asked why the corn and herbs are added, people invariably respond "*mihunapaq*" or "*para alimentacion*" ("for food," "for nutrition"), although the nutritional value of such small quantities of corn, in contrast to the salt, would seem relatively minor for a herd of any size. More likely, it serves as a medium for distributing the salt. Insufficient salt can itself lead to decreased milk production [42]. Supplements with animal protein are infrequent, and the small quantity (no more than a kilogram or two), is distributed throughout the herd. My host family usually prepared two piles of the salt-corn mixture, one for the cows, the other, without the flicker supplement, for the bulls, but I don't know how common this practice is.

While ruminants are better able to take advantage of most plant protein than humans, protein supplements from animal sources – fishmeal, bloodmeal, feathermeal – are used in the dairy industry in industrialized societies [42,43]. Protein metabolism in dairy cows is complex and the mechanisms are not well understood. While undegraded feed protein is important for milk production, the effect of animal-based supplements with a naturally low protein degradation rate is still not fully-known [43]. Most studies have been conducted on high-yielding commercial cattle breeds rather than on the *criollo *varieties raised by subsistence farmers in developing countries, creating problems of comparability.

It seems likely that the occasional use of fishmeal in Ccachín, a product that must be purchased, was originally learned through peonage on the haciendas prior to agrarian reform or from extension agents from the Ministry of Agriculture. The practice of giving protein supplements in general may be explained in terms of the adoption of Western animal husbandry practices, with the lack of economic resources accounting for the near homeopathic quantities given in most cases.

Cattle are not indigenous to the Andes, and many husbandry practices accompanied their introduction by the Spanish. Fennel, for example, one of the herbs added to the corn-salt mix given to cattle in Ccachín, is indigenous to the Mediterranean region, and both the spread of the plant to the Andes and its use as a galactagogue in the region are the result of diffusion. On the other hand, the use of *hak'achu *as a galactagogue for humans is pre-Columbian in origin and thereby predates the introduction of cattle to the Andes. What's more, neither of the two domesticated herd animals native to the Andes – the llama and alpaca – were bred to be milked, making the Inca and their predecessors dairyless civilizations [44]. Thus, the ethnoveterinary use of the bird as a galactagogue in dairying postdates the Inca empire. Whatever role the diffusion of western animal husbandry practices plays in the giving of protein supplements to dairy animals, it does not explain the specificity of using the *hak'achu*.

### Sympathetic caring for the herds

Where might the explanation lie? There seems to be a strong element of sympathetic caring for the herd, with relations patterned, as with the local landscape and other living creatures, along the lines of human social relations. In *aqhata uhuchiy*, herders in Q'ero and other highland Peruvian communities ritually bottle-feed corn beer to their llama soon after transporting corn from fields in the lower valley, sharing with them the prestigious product of their joint labor [45]. In Ccachín, corn beer is considered to be medicine for the llama and alpaca, and households set aside surplus corn beer from social and ritual festivals to be given to their herds. Likewise, coca leaves – the sharing of which is essential for creating and maintaining harmonious social relations among people and respectful relations with mountain spirits – are placed in the mouths of sheep, goats, and other animals in Ccachín as an offering before they are butchered.

Beer is not only used in the Andes as a galactagogue by women, it is used as such in many places around the world, both for humans and animals. Along with fermented and sprouted sorghum seeds, for example, beer is used by the Dinka, Nuer, and other East African peoples to boost milk production in their herds [40].

In a study of salt ball feeding in the southern Ecuadorian highlands, Hirschkind has shown how the giving of salt to cattle is itself metaphorically patterned on human attributes and relations [38]. Salt ball feeding is part of a general approach called *estimando el ganado *(esteeming the cattle) in Rivera Parish (Cañar Province), her research area. Just as salt, lard, and sugar are fundamental ingredients in the local cuisine, they are measured out, formed into balls, and served individually to the cattle on a regular basis by those who can afford to. The biological, nutritional, and psychological attributes of cattle are modeled on the corresponding human attributes. Humans and cattle are believed to share similar health problems, the hot-cold categories used to classify human illnesses and cures are applied to cattle as well, and the same herbal teas, topical ointments, and pharmaceuticals are given to both. Parallel to the serving of flicker flesh to both lactating women and cattle in Ccachín, guinea pig soup is used as a galactagogue for both women and cattle in Rivera, Ecuador.

Such modeling of animal characteristics and care on human qualities and social relations is by no means limited to the Andes [46]. Whatever the origins of the specific association of the Andean flicker with milk production, serving it to both humans and domestic animals as a galactagogue fits well with a general tendency in the Andes and elsewhere to replicate human relations in the treatment of valuable livestock.

## Discussion and conclusion

Galactagogues provide an especially rich and provocative research topic for anthropologists because they conjoin practical and symbolic logics, and because the very belief that a given treatment is effective often makes it so for humans, whatever the original reason for its adoption. The metaphorical associations not only substitute relations of likeness for likelihood, they have the potential to blur the boundaries between them. In this sense, they have the same subversive quality that Anne Harrington notes for placebos: "For me, placebos were a wonderfully subversive phenomenon in the world.... They seemed to function [in] narrative, symbols, human relationships, and other things thought about in the humanities and social sciences, but they also happened in the body, and looked a lot like biology" [quoted in [47]].

### The origin and transmission of Andean galactagogues

Given the wide range of potential functional equivalents when such psychophysiological processes are at play, explanation of the origin and survival of particular practices turns on historical contingency and cultural selection processes. In terms of the cultural belief system as a whole, they may be anything but arbitrary. But with origins in antiquity, the original motivations and meanings for many Andean galactagogues are difficult to determine. The possibilities I have identified here – color associations, the dry/wet contrast and dessication/reconstitution, salient features of the items that relate them to lactation, associations with opening up, tenderizing and softening, or spilling forth – all find metaphorical expression in the wider cultural context and merit further research.

Once established, the proximate cause of an item's use as a galactagogue may be some kind of learning bias – following the crowd or following the lead of prestigious individuals – rather than the associations themselves, which are rarely articulated. These, in fact, are the reasons most often given by the practitioners themselves. The standard answer I received to my questions about why a particular treatment was used in a given context in Ccachín did not make reference to any underlying principle, it was invariably a reference to authority: "*mi madre sabe como*..." ("my mother knows how to..."), "*los viejitos saben*..." ("the elders know..."), "*los machulas lo usaron*..." ("the ancestors used it..."). In each case, the assumption that the treatment is effective is reinforced by the popularity of its use or the general success of its users, rather than determined empirically from its use itself. Notably, the most common reason villagers give for doing something in ceremonial and ritual contexts – "*por costumbre*" ("by custom") is not invoked to explain ethnomedical practices. Tradition alone may be taken as sufficient reason for doing some things, but not when one's health is at stake.

In drawing upon the biodiversity of their environment, indigenous people are faced with the same problem in filling out their medicine chest as Western medical science is, that is, "Of the vast and bewildering variety of organisms available, which ones should be collected and analyzed?" [48]. One solution to the problem is to use some sort of cultural screen. In the Andes, metaphorical associations and hot/cold food concepts are the most common. The animal, mineral, and plant resources identified in this way, along with those suggested serendipitously, by their similarity to known curatives and other processes, pass through a second screen – attending to outcomes – which allows through those items with perceived benefit, whether that benefit be by pharmacological or psychosomatic effect.

Another solution to sorting through the vast variety of possibilities offered by the natural world, is to follow the lead of others who have done the screening already. This can be seen in the number of galactagogues that have diffused to the Andes, most long ago from Europe. This is often the approach of naturopathic health practitioners in the West as well. Genetic prospectors from the pharmaceutical industry likewise turn to the reservoir of knowledge of indigenous peoples for guidance about which plants are worth taking a closer look at. Once in the lab, these items are subjected to a third screen, that of controlled experimentation.

### Practical and symbolic logic

In *Coevolution: Genes, Culture and Human Diversity*, anthropologist William Durham argues that symbolic reasoning is a fundamental organizing principle of cultural systems, but that the sphere in which such reason has free play in relatively limited. As he puts it,

"I would expect cultural reason to be truly arbitrary and nonpractical only to the degree that the variation on which it acts is, or is perceived to be adaptively neutral. Among neutral variants in language, meat-cut preferences, art or fashion, arbitrariness may well be the general rule... But there is certainly nowhere near the same degree of arbitrariness in the marriage principles of Tibet, the milk-use customs of Europeans, and the incest taboos of the world probability sample"[49].

Given the importance of breast-milk for infant survival, it would seem to fit the first group of practices discussed by Durham more than the second, and indeed, natural selection itself has assured that lactation failure is physiologically rare. But fear of failure is not as rare as failure is, and the role that anxiety plays in inhibiting milk secretion, along with the beneficial role that galactagogues can play as confidence inducers to reinforce the let-down reflex, allows for cultural selection among a wide range of culturally-salient but pharmacologically-neutral alternatives. In fact, to the extent that placebo effects and similar psychological processes are important to the success of many kinds of health treatments, the potential role of cultural selection is larger in this domain than Durham implies.

If Durham is right on the larger issue, however, most maladaptive nursing practices are likely to be eliminated through a combination of empirical observation and natural selection over time. The World Health Organization Guidelines for the Assessment of Herbal Medicines assume as much in saying that "Prolonged and apparently uneventful use of a substance usually offers testimony of its safety," and establishes that "when there are no detailed toxicological studies, documented experience of long-term use without evidence of safety problems should form the basis of the risk assessment" [50].

As far as I'm aware, none of the galactagogues used in the Andes as listed in Table 1 are counterproductive in this sense. Rather, they are either neutral in terms of their direct effect on health (e.g., the stone amulet, and the plaster of sweet potato eyes), or far more commonly, conducive to general health (e.g., the various beers, broths, soups, and meats), though not likely therapeutic in the quantities served. Where this conclusion may be open to question – as in Paratía (Puno, Peru), for instance, where women who have recently given birth are urged not to use salt in their food [51] – insufficient information on background conditions and intake levels is available to determine the overall health effects.

To say that there should be a tendency for maladaptive nursing practices to diminish over time, however, is not to suggest that all nursing practices that are harmful to infants will tend to disappear. Adaptation is defined here from the perspective of the parents, not the children, and their respective interests differ. McKee, for example, notes that restricted female access to breast milk is reported for several areas of the Andes [52]. In the Ecuadorian highlands, differences in the length of the nursing period for boys and girls are sustained by the belief that sexuality and aggression are transmitted from mothers to daughters during nursing, so that if a girl is nursed past a year, she will become licentious and rebellious. In the mestizo village of Ecuador where McKee conducted research, girls were weaned on average at 10.93 months, boys at 20.27 months, with 8.25 and 24 months being the cultural ideal for weaning of girls and boys respectively. Early weaning is implicated in severe diarrhea and increased infant mortality. McKee argues that while the physiological and economic costs of such practices are high relative to the alternative ways that parents have for regulating the number of their offspring, the psychological and social costs of the other strategies may be even greater.

According to Baumslag, many of the dietary restrictions on lactating mothers in developing countries around the world are counter to the recommendations of scientific medical practice [10]. In light of the above, however, before we conclude that the scientific community is wrong about the particulars in these instances, or that the area of maternal health is one where cultural practices are more maladaptive than one might expect, the conflicting interests of the various decision makers with responsibility for a newborn's welfare must be taken into account.

### The conceptual blending of human and domestic animal domains

The use of the Andean flicker – *hak'achu *– as a galactagogue likely first emerged as a treatment for nursing women, and only later became applied to animals as well, at least with regard to dairying. In areas where cattle-raising became part of the post-conquest rural subsistence strategy, a positive feedback loop emerged, with the ethnomedical treatments sympathetically extended to dairy herds, and the observed geographic association of *hak'achu *with their status as a sentinel and protector extending backwards from cattle to humans, reinforcing the association.

Hirschkind indicates that hot/cold food concepts are applied equally to humans and animals in Ecuador [38], and McCorkle and Martin report that in most cultures, ethnomedical practices are generally the same for animals and people, including not only healthcare concepts and beliefs, but specific ethnopharmaceutical materials, treatment techniques, behavioral controls, and medico-religious interventions [53]. They further note that " [I]n all ethnomedical systems, special foods, beverages, or feedings are routinely prescribed to treat or prevent various conditions in both livestock and people. Such prescriptions are universally popular for problems of fertility, libido, pregnancy, lactation, appetite, and energy, for instance" [53]. The ready transfer of ideas across ethnomedical and ethnoveterinary realms in the Andes [38,54], as elsewhere, is in itself sufficient to explain the use of Andean flicker carcasses as a galactagogue in both contexts.

Coincidently, the contemporary use of animal proteins such as *hak'achu *flesh and crayfish to improve the milk production of cattle in the area is further reinforced by local attempts to emulate the use of feathermeal and fishmeal in the commercial dairy industry as well, and indeed, the few who can afford it may use the commercial products in their stead. In terms of effectiveness, whatever beneficial psychosomatic effect that *hak'achu *flesh and other galactagogues have among human populations is lost when the same practices are transferred to livestock. The benefit of following Western dairy science in using animal-protein supplements to enhance milk production is likewise lost when transferred to the rural Andean setting, where the typical farmer does not have the resources to employ them in sufficient quantity to do any good. The ethnoveterinary use of *hak'achu *is not explainable in terms of its reputed beneficial effects, nor as the outcome of trial-and-error learning, natural selection of adaptive cultural traits, or other processes that invoke such effects. Instead, it likely represents the transfer of a practice that can be observed to be beneficial in the human realm to domestic animals by analogy, reinforced by attempts to imitate more recently introduced Western animal husbandry practices.

## Conclusion

I have discussed the therapeutic use of Andean flickers in a Peruvian village along with the wide variety of galactagogues used in the Andes, and I have attempted to identify some common patterns and principles in their use. My aim has been to define the scope and potential interrelationship of the factors involved in Andeans' use of flickers as a galactagogue. The use of the Andean flicker raises important questions about how people perceive differences between likeness and likelihood, psychological and pharmacological effects, and health care in human and nonhuman animal domains. The Andean flicker and other galactagogues used in the area provide a fascinating vantage-point for further exploration into the principles underlying traditional Andean pharmacology and zootherapy.

## Declaration of competing interests

The author(s) declare that they have no competing interests.
